# The role of severity perceptions and beliefs in natural infections in Shanghai parents’ vaccine decision-making: a qualitative study

**DOI:** 10.1186/s12889-018-5734-9

**Published:** 2018-06-28

**Authors:** Xiaodong Sun, Zhuoying Huang, Abram L. Wagner, Lisa A. Prosser, Erzhan Xu, Jia Ren, Bei Wang, Wenlu Yan, Brian J. Zikmund-Fisher

**Affiliations:** 1 Department of Immunization Program, Shanghai Municipal Centers for Disease Control & Prevention, 1380, West Zhongshan Road, Shanghai, 200336 NO China; 2 Department of Epidemiology, School of Public Health, University of Michigan, 1415 Washington Heights, Ann Arbor, MI 48109 USA; 3 Child Health Evaluation and Research Unit, Department of Pediatrics and Communicable Diseases, University of Michigan Medical School, Ann Arbor, MI USA; 4 Department of Health Behavior & Health Education, School of Public Health, University of Michigan, 1415 Washington Heights, Ann Arbor, MI 48109 USA; 5 School of Information, University of Michigan, 4322 North Quad, 105 S. State St, Ann Arbor, MI 48109 USA; 6 Department of Internal Medicine, Division of General Medicine, University of Michigan Medical School, 1500 East Medical Center Drive, Ann Arbor, MI 48109 USA

**Keywords:** China, childhood vaccination, vaccine hesitancy, qualitative study, individual interviews, decision-making

## Abstract

**Background:**

China has reduced incidence of vaccine-preventable diseases through its Expanded Program on Immunization (EPI). Vaccines outside of the EPI are not provided for free by the government, however. This study explored how the stated importance of different disease and vaccine-related attributes interacted with beliefs about the immune system of a child to affect Chinese parents’ decision to obtain a non-EPI vaccine.

**Methods:**

Mothers and fathers of young children at immunization clinics in Shanghai, China, were interviewed about vaccine decision-making and what attributes of a disease were important when making this decision. An inductive thematic analysis explored their beliefs about disease attributes and how these related to vaccination decisions.

**Results:**

Among the 34 interviews, severity of the disease—particularly in causing long-term disability—was the most commonly cited factor influencing a parent’s decision to get a vaccine for their child. Many parents believed that natural infection was preferable to vaccination, as long as disease was not severe, and many were concerned that imported vaccines were inadequate for Chinese children’s physical constitutions. All these beliefs could influence the decision to vaccinate.

**Conclusions:**

Many parents do not appear to understand how and why vaccines can support development of a healthy immune system. Because severity emerged as parents’ overriding concern when making decisions about vaccines, marketing for a childhood vaccine could focus on the severe condition that a vaccine can protect against.

## Background

Vaccines have been an instrumental in reducing the burden of infectious diseases throughout the world, including in China [1, 2]. At the same time, the general population in many countries has expressed a variety of questions and concerns about vaccines. Although vaccine hesitancy is a global phenomenon, its manifestation differs across countries and regions; efforts to promote vaccine confidence within a local population should focus on the specific skepticisms and beliefs present in this local population [3].

China has a two-tiered vaccination program, whereby category 1 vaccines are provided for free by the government to citizens throughout the country at immunization clinics and category 2 vaccines are available for a cost at these same clinics. The China Experts Advisory Committee on Immunization Program decides which vaccine candidates are to be listed as category 1 vaccines within the Expanded Program on Immunization (EPI). Proof of prior administration of EPI vaccines is required for school entry, and coverage of these vaccines is therefore typically high throughout China, and especially in wealthier cities in Eastern China. After approval from the China Food and Drug Administration, other vaccines, such as rotavirus, *Haemophilus influenzae* type b (Hib), and pneumococcal conjugate vaccine (PCV) can be sold and administered at immunization clinics as category 2 (non-EPI) vaccines [4]; these vaccines are administered according to manufacturer’s instructions or ad hoc policies developed at local immunization clinics and are not subject to the purview of the Experts Advisory Committee on Immunization Program. EPI vaccines produced by foreign manufacturers are also available for sale at immunization clinics and are considered category 2 vaccines.

Because of their cost and because they are not required for school entry, category 2 vaccines have lower coverage than category 1 vaccines. In a survey of electronic vaccination records from Guangzhou, only about half of children were vaccinated against rotavirus by 5 years of age [5]. A study from the Shanghai immunization information system found that, among children between the ages of 2 and 6, 50.9% had received Hib and 11.4% had received PCV. A separate factor that contributes to lower coverage is that both vaccines were typically delayed so as not to be co-administered with category 1 vaccines [6].

Many previous qualitative and quantitative studies in Western countries have explored how parents decide to give their child a vaccine [7]. Vaccine decision-making can be informed by attributes of the disease (e.g., severity of the disease or likelihood of acquiring it), and these beliefs can be shaped by parents’ knowledge of the immune system [8, 9]. Lack of trust in health care professionals may also lead parents not to immunize their child [10].

Studies on vaccine decision-making in China are limited. Previous qualitative studies have examined vaccination decision-making among Chinese mothers in Hong Kong [11], and hypothetical acceptance of a pediatric and adult Shigella vaccine in mainland China [12]. What attributes are important for parents in mainland China, however, have not been thoroughly explored previously. Given China’s status as a middle-income country and because of the two-tier structure of China’s vaccination program, Chinese parents may be subject to different influences or process health care decisions differently compared to Western parents. Through qualitative, in-depth, individual interviews, this study summarizes how parents discussed the disease- and vaccine-related attributes that they perceived as important when making a decision about a vaccine, and details how these attributes and beliefs about the immune system of a child affected parental vaccine decision-making. The attributes identified in this study can be used to inform the development of quantitative questionnaires, and these topics can be emphasized in marketing and educational materials that promote vaccinations.

## Methods

### Study design

This qualitative study was based on semi-structured, individual interviews. The interviews took place in Shanghai, China, a municipality of 24 million people in Eastern China, which has emerged as an important destination for millions of migrants, or *non-locals*, from other provinces in China. In the summer of 2016, caregivers accompanying young children at immunization clinics in Songjiang, Baoshan, Huangpu, Changning, and Minhang districts were asked for their interest in participating in the study. The potential participants were selected from waiting rooms after their child had received a vaccine. If they expressed interest, they were given an informed consent form to read and to sign their agreement to participate and to be recorded. The actual interview took place in a private room at the immunization clinic and was recorded. Data collection was concluded after saturation occurred, meaning no significantly new material was gained in the previous 3 interviews.

### Data collection

The questions were inspired by previous qualitative research in Western countries [13–17]. Questions were open-ended, and responses were probed to explore the participants’ thoughts and to discover situations and attitudes unique to the Chinese context. Because our main aim was to explore vaccine decision-making, we first had parents discuss their thoughts on free, EPI vaccines, and how those thoughts contrasted with vaccines they would have to pay for. Because all current EPI vaccines are produced in China, whereas non-EPI vaccines could be produced in China or abroad, participants were also asked if the location of vaccine manufacturer affected their willingness to obtain a vaccine. Receipt of non-EPI vaccines could mean that a child receives more vaccines at an earlier date, and previous research suggests that some Shanghai parents have substantial reservations about co-administering vaccines during the same visit, and administering multiple vaccines to infants at a young age [18]. Together, these two concerns are termed “vaccine scheduling.” Finally, based on findings from another quantitative study in Shanghai [19], we were interested in how perceptions of disease severity contrasted with disease susceptibility in a parent’s decision to obtain a vaccine. Table 1 lists the initial questions used in the interview.

**Table 1:** List of interview questions, Shanghai, 2016

1. Tell me a little bit about your thoughts on childhood vaccination.	
2. The governments of some countries mandate children to be vaccinated while some don’t. What do you think of that?	
3. Some vaccines at the clinic are free and some are for-fee. Can you walk me through how do you decide whether or not to give your child a voluntary, for-fee vaccine?	
4. Can you help me understand what influences people’s decision to vaccine their child with a for-fee vaccine?	
5. What do you know about pneumonia and pneumonia vaccines?	
6. What do you know about meningitis and meningitis vaccines?	
7. How important is the manufacturer in your vaccine decision? Specifically foreign vs domestic manufacturers?	
8. Does what you hear in the news influence your decision to get a vaccine?	
9. What about the number of vaccines a child receives in the first year of life, do you think it is too much, about right, or too few? Why?	
10. Think about a situation where your child needed to get 5 different vaccine shots within two months. You can decide to get these all in one visit, or to come to the immunization clinic multiple times. Ideally, how often would you want to come to the clinic, and how many shots would you like to be given to your child in one visit?	
11. What is the most important characteristic about a vaccine that would or would not make you want to give the vaccine to your child?	
12. What is the most important characteristic about a disease that would or would not make you want to protect your child against the disease with a vaccine?	

### Analysis

The interviews, conducted in Mandarin Chinese, were transcribed by a native Mandarin speaker and translated into English. Qualitative coding was done on the English transcriptions, with frequent references to the Chinese. Thematic analysis [20] was used to explore issues surrounding vaccine decision-making. The interviews were coded by two researchers in an inductive process, in which the codes and themes were found based on what was present in the data, although they were informed by concepts (such as susceptibility and severity) from the Health Belief Model. Initially, codes were developed based on distinct ideas and events found within an interview. Subsequently, similar ideas across interviews were coded together. These codes were then collated into larger, internally-consistent themes. The interviews were analyzed in NVivo 10 (QSR International, Melbourne, Australia).

## Results

Of 44 parents of young children approached to be in the study, 10 parents refused to participate, and 34 completed the in-depth, structured interviews: 9 fathers and 25 mothers (Table 2). They came from eight different districts of Shanghai: 13 were from districts in the urban, inner city of Shanghai (6 from Huangpu, 5 from Changning, and 1 each from Jing’an and Putuo), 11 were from districts in the inner suburbs (7 from Baoshan, 4 from Minhang, and 1 from Pudong), and 9 were from Songjiang, a district in the outer suburbs. Half (17) were locals, with an official residency in Shanghai, and the other half were non-locals and had an official residency from outside of Shanghai. Twelve had a second child.

**Table 2:** Characteristics of participants in study, Shanghai, 2016

	Count	Proportion
Age (in years)		
23-26	6	18%
27-30	10	29%
31-34	11	32%
35 and above	6	18%
Relation		
Mother	25	74%
Father	9	26%
Residency		
Local	17	50%
Non-local	17	50%
Urbanicity		
Urban	13	38%
Outer suburbs	11	32%
Inner suburbs	9	26%
Number of children		
1	22	65%
2	12	35%

Table 3 lists representative quotes from the participants and related themes. Participants were divided in their beliefs about the utility of (mandatory and free) EPI vs non-EPI vaccines, especially as administration of additional non-EPI vaccines would add to their child’s vaccination schedule. Several disease-related attributes that could affect vaccine decision-making emerged with varying degrees of intensity but could be categorized into either focusing on disease severity or susceptibility. Several parents also believed that their child’s immune system negated the need for certain vaccines, or that natural infection was better than vaccination to induce immunity. In regards to the vaccine itself, some parents believed that the effectiveness of the vaccine could be influenced by their child’s “physical constitution,” and that there could be differences between foreign and domestic vaccines in terms of vaccine effectiveness or safety.

**Table 3:** Distribution of different themes arising from in-depth interviews about vaccine attitudes, Shanghai, 2016

Topic	Theme	Number affirming response	Representative quotes
Mandatory and free vaccines	Children should only get mandatory vaccines and no optional vaccines.	5/34	“We are basically doing our vaccines according to the card. […] If it says something is due, we will just do it. Nothing more.” (Participant 20)
	For-fee vaccines are a conflict of interest for immunization clinics.	3/34	“I think that for all of the for-fee vaccines there must be some conflict of interest. And the quality of vaccines is vital to children… Like the vaccine scandal last time, right? And the free vaccines […] are implemented nationwide for all children. They shouldn’t have any big problems. Moreover, there shouldn’t be any conflict of interest involved.” (Participant 24)
Vaccine scheduling	Co-administering multiple vaccines in one visit causes too much pain for the child.	8/34	“Of course I will do one shot at a time! Doing five shots at once is too terrible! […] If you do multiple shots, that would be too much pain, right? She will cry every time, and will not get settled afterward.” (Participant 7)
	Combination vaccines are convenient by limiting the number of times families must come to clinic.	4/34	“For example the pentavalent vaccine we got today. It saves many office visits and shots for my child.” (Participant 32)
Severity	Mild diseases do not require vaccination	4/34	“For example, diseases that only need a few days resting at home, I think, we may or may not get those vaccines.” (Participant 3)
	Diseases impacting development or brain function should be targets of vaccination.	9/34	“For diseases that need hospitalization, or will lead to severe outcome like disability, we definitely will get vaccines.” (Participant 13)
			“Many vaccines in this country are […] mandatory. This maybe is due to fact that the disease is fatal, or it will have severe impact on, for example, your ear development or anything. So those are necessary. And I wouldn’t necessarily consider [vaccines] for a disease like the pneumonia or something like that.” (Participant 11)
	Severity is the most important disease attribute to consider when making a decision to get a vaccine.	11/34	“It is the severity, not the prevalence, but the severe outcome which is more important. So I will normally consider getting a vaccine for a severe disease.” (Participant 16)
Susceptibility	Prevalence is the most important disease attribute to consider when making a decision to get a vaccine.	7/34	“Like chicken pox, this is necessary. Because the prevalence is quite high. Even if it is a voluntary vaccine, I will still get it for this price.” (Participant 17)
	Children should receive vaccines to protect against diseases easily transmitted.	6/34	“Because you will never know where you might encounter disease. Moreover, nowadays, many parents are bringing their kids to rural areas where insect-borne disease are possible. Respiratory infection, insect-borne diseases could all be encountered. So I will get my children vaccinated, regardless of the chance. I have to prevent that.” (Participant 2)
	Contagiousness is the most important disease attribute to consider when making a decision to get a vaccine.	5/34	“Maybe diseases that are highly contagious also need to be vaccinated. Not only does it harm the kid, but others as well.” (Participant 3)
Immune system and physical constitution	Immunizations are needed when or if the immune system is not adequately developed.	3/34	“I took my son to get a flu shot when he was one year old. Because I think my kid at one year old does not have a well-developed immune system. [..] Then we also got that when he is two. But when he turned three years old, we stopped. I think his immune system will be better since he has had exposure to more diseases.” (Participant 6)
	The immune system works fine by itself, and so some immunizations may be unnecessary.	7/34	“Because for pneumonia you only need to take the [intravenous] drip to get better. There is not a large consequences [for disease]. And for the kid, getting sick once in a while isn’t that bad of a thing, as long as the child can recover from it and be treated for it.” (Participant 11)
	Contracting a disease is mediated by an individual’s physical constitution.	10/34	“For me I sometimes think it is not necessary to vaccine on purpose. In fact, for meningitis or any inflammation, it is caused by low levels of immunity in the child and low quality nutrition. In general, changes in the physical constitution will cause these inflammations.” (Participant 24)
			“I have two children in the family who developed [pneumonia]—my brother and sisters’ kids. So I felt this vaccine is necessary! Very terrible. But later, based on my child’s physical constitution, and his history in resisting flu or other viruses, we decided not to get this shot.” (Participant 18)
			“We chose not to get the Hib vaccine because I think my child’s immunity is okay. Nor did we get the pneumonia vaccine.” (Participant 6)
	Too many vaccines are harmful to an individual’s immune system (particularly for those with a certain physical constitution).	6/34	“If a child has an allergic physical constitution, I mean, generally speaking, allergies don’t bother people too much. But other people have a physical constitution with an extreme sensitivity to allergies. Then if they get too many vaccines at once, I am just concerning that they can’t handle them physically.” (Participant 21)
Location of vaccine manufacturer	Chinese-made vaccines are more appropriate.	12/34	“When comparing Chinese vaccines to foreign vaccines, how do you know if the imported ones are more suitable to the physical constitution of Chinese babies?” (Participant 3)
			“So if [the vaccines] are produced by the country, there shouldn’t be any problem. But it is not necessarily the case when it comes to for-fee vaccines. […] I will not get imported vaccines, or other imported products like baby formula, because the physical constitution of foreign children are different than ours.” (Participant 24)
	Foreign-made vaccines are more appropriate.	16/34	“I had a problem when my daughter got the vaccine, who had a big reaction after getting the country-provided vaccine, but who has had very little reaction after getting the imported ones. […] My daughter got the domestic vaccine once, and she vomited right after the shot, right here. […] So I always chose the imported vaccines after that.” (Participant 10)

### Mandatory and free vaccines

In regards to the difference between mandatory (EPI) vaccines and non-mandatory (non-EPI) vaccines, some parents only received vaccines that were mandatory, and did not opt to pay for the additional vaccines. “We are basically doing our vaccines according to the card. […] If it says something is due, we will just do it. Nothing more” (Participant 20). For a few parents, this decision to only receive mandatory vaccines was based in part on a belief that doctors had a financial conflict of interest when they promoted for-fee vaccines.

Conversely, for a few participants, all vaccines were important, and the parents would choose for their child to receive many vaccines.. “I will get the vaccine regardless of how severe the disease is. […] I want comprehensive protection, so I think I should get everything” (Participant 5). This mention of “comprehensive protection” seemingly values more vaccines over fewer, although protection from the vaccination schedule is of course limited by imperfect vaccine efficacy.

### Vaccine scheduling

A few parents stressed the logistical advantages of getting combination vaccines, such as the pentavalent vaccine, which is an optional, non-EPI vaccine. “For example the pentavalent vaccine we got today. It saves many office visits and shots for my child” (Participant 32).

Preference for combination vaccines (and conversely, aversion for co-administering multiple vaccines during one office visit) was related to parents not wishing to see their child suffer through multiple injections “Of course I will do one shot at a time! Doing five shots at once is too terrible! […] If you do multiple shots, that would be too much pain, right? She will cry every time, and will not get settled afterward” (Participant 7). Parents expressed a strong preference for co-administering as few vaccines during one visit as possible (commonly a maximum of two).

### Severity

Most participants qualified their support for obtaining an optional, non-EPI vaccine based on some attribute of the disease the vaccine protected against. For many respondents, severity (and not measures of disease susceptibility, such as prevalence of disease or contagiousness) emerged as the most important disease attribute to consider when making a decision about a vaccine.

For several parents, the severity of a disease could be quantified by how long the child would be sick, or if the disease would result in disabilities or long-term effects: “For example, diseases that only need a few days resting at home, I think, we may or may not get those vaccines” (Participant 3). In this respect, duration was an important aspect of disease severity.

One parent brought up how disease could disrupt a child’s academic schedule and the family’s life: “The treatment [for pneumonia] might require hospitalization, because they need to monitor and observe the patient. That means at least two weeks of school absences. My daughter got the disease right after her final exam, so the effect was minimum. But if it happened before her exam period, it could be a huge impact. But even in her case, we stayed at the hospital and kept her company at all times. Our family routine was interrupted. That affected our lives and work” (Participant 2).

Other parents mentioned how they were particularly concerned about diseases that affected the brain or led to development issues. “Well I know meningitis, if you recover from meningitis, than it will be fine. But if you don’t, it will affect you in many ways” (Participant 1). For this parent, disease severity was related to the potential for long-term sequelae.

### Susceptibility: prevalence and contagiousness

Beyond disease severity, another disease-related attribute that parents were asked about was susceptibility. This was split into two dimensions: prevalence and contagiousness. A few parents thought that vaccines are necessary based on the prevalence of disease. “Like chicken pox, this is necessary. Because the prevalence is quite high. Even if it is a voluntary vaccine, I will still get it for this price” (Participant 17).

Overall prevalence of disease was not as important of a consideration as the contagiousness and mode of transmission of the disease, which more often influenced parents’ decisions on whether or not to obtain a vaccine. “Because you will never know where you might encounter disease. […]. Respiratory infection, insect-borne diseases could all be encountered” (Participant 2). Therefore for this participant, infections which could be transmitted in a relatively public setting should be prioritized for vaccination.

Contagiousness was most often conceived of as a way that a disease could affect one’s own child, but a few parents also brought up that by having their child vaccinated, they could protect others. “Maybe diseases that are highly contagious also need to be vaccinated. Not only does it harm the kid, but others as well” (Participant 3).

### Immune system and physical constitution

Many parents discussed their belief that being immunized with a vaccine impacted the development of their child’s immune system. Some parents mentioned that vaccines are particularly necessary for when the child is young and has an immature immune system: “I took my son to get a flu shot when he was one year old. Because I think my kid at one year old does not have a well-developed immune system. [..] Then we also got that when he is two. But when he turned three years old, we stopped. I think his immune system will be better since he has had exposure to more diseases” (Participant 6).

Some parents expressed the belief that having a natural infection was preferable to vaccination or at least did not have large consequences, as long as it was not a severe disease: “So if it is like normal headache or cold, they can just recover by themselves if they have good immunity. They don’t need vaccines” (Participant 30).

Many parents brought up the concept of physical constitution (tizhi 体质) in describing their beliefs about vaccinations or immune responses to disease. Several parents believed how the physical constitution of a child could be improved without needing vaccines. “For me I sometimes think it is not necessary to vaccine on purpose. In fact, for meningitis or any inflammation, it is caused by low levels of immunity in the child and low quality nutrition. In general, changes in the physical constitution will cause these inflammations” (Participant 24) .

Parents brought up physical constitution to describe variations in if a child needed a vaccine or not. “There are some kids who are one year older than my son. They all came down with the same problem, hospitalization due to pneumonia. So back then I was very nervous, because I thought it was very severe, like persistent fever, so of course I was very nervous. Then my child was just born. I have two children in the family who developed [pneumonia]—my brother and sisters’ kids. So I felt this vaccine is necessary! Very terrible. But later, based on my child’s physical constitution, and his history in resisting flu or other viruses, we decided not to get this shot” (Participant 18).

### Location of vaccine manufacturer

Several parents believed that the physical constitution varied between Chinese children and children from other countries. Some were concerned that these differences could impact effectiveness of the vaccine, so that Chinese-made vaccines were more effective for Chinese children than foreign-made vaccines. “When comparing Chinese vaccines to foreign vaccines, how do you know if the imported ones are more suitable to the physical constitution of Chinese babies?” (Participant 3).

There was approximately a 50-50 split between parents preferring domestic versus imported vaccines. Preference for foreign vaccines was often tied to safety issues: “I had a problem when my daughter got the vaccine, who had a big reaction after getting the country-provided vaccine, but who has had very little reaction after getting the imported ones. […] My daughter got the domestic vaccine once, and she vomited right after the shot, right here. […] So I always chose the imported vaccines after that” (Participant 10).

## Discussion

Understanding the formation of vaccine beliefs, and how perceptions of a vaccine or disease can influence an individual’s decision to get a vaccine, underpins research in vaccine hesitancy [21]. Some vaccines in China are both free and mandatory for school entry, but many are not. This study focused on what parents thought of vaccines in general and how they decided to obtain for-fee, voluntary vaccines. We identified severity as the predominant disease attribute affecting parents’ decisions to give their child an optional vaccine. Parents framed their vaccine decision-making within inter-related and larger conceptions of their child’s immune system, notably whether vaccination or natural infection was more appropriate to gain immunity, how a child’s physical constitution predisposed them to disease, and whether domestic or imported vaccines were better suited for a Chinese child.

### Disease attributes

Perceived severity of disease was important for participants in this study; caregivers were, in general, more likely to want a vaccine against a severe disease than a more common disease. Wang et al.’s qualitative study of Chinese mothers in Hong Kong also found that the seriousness of diseases that were prevented by mandatory vaccines legitimized vaccination [11]. Quantitative studies have also born out the importance of severity. For example, during the 2009 H1N1 influenza pandemic in Australia, higher perceived severity of H1N1 was associated with 2.5 times higher odds of acceptance of vaccination compared to those who perceived H1N1 to be less severe [22]. Brewer et al. conducted a meta-analysis of 34 studies (mostly from the US and other developed countries) and found that greater perceived severity was associated with increases in vaccination behaviors [23]. Severity of disease among our participants was often thought of in terms of long-term disability or damage to the brain; framing certain vaccine-preventable diseases in terms of these disabilities could be a reference point for doctors wishing to encourage vaccination.

Brewer et al.’s study also found that increases in perceived likelihood of disease was associated with vaccination behaviors [23]. Some parents in our study were concerned about diseases perceived of as common, like chickenpox, but for many it was not the predominant attribute that they considered when thinking about a vaccine-preventable disease. They framed their perceived risk of getting a disease in terms of how contagious the disease was, which was tied, for some, in how the disease was spread (for example, respondents brought up respiratory diseases as a particularly contagious disease).

### Physical constitution

A highly prevalent belief was that the physical constitution of a child could affect their receptivity to vaccines. Previous studies have investigated the phenomenon of physical constitution (alternately translated as body or personal constitution) in East Asia, although these mainly focused on characterizing distribution of various manifestations of physical constitution and its relation to chronic disease in adult populations [24, 25]. Although the respondents in our study referred to physical constitution in an abstract sense, it derives from standard clinical practices within traditional Chinese medicine which seek to predict a patient’s propensity for disease or health based on imbalances within the body or between the body and environment [26]. That parents are concerned about vaccine responses based on their perception of their child’s physical constitution is a novel finding and has implications for how doctors should recommend vaccines.

### Perceptions of Chinese-made vaccines and the Chinese health care system

Some of the parents in this study preferred domestic vaccines (often because they believed Chinese-made vaccines accorded better with a Chinese child’s physical constitution), but others preferred imported vaccines. These preferences are tied to perceptions of vaccine quality. Quality of pharmaceutical and food products may be a particular concern for parents in China, where counterfeit products are widespread and have been linked to serious illness in children and hospital patients [27–29]. A qualitative study of Chinese mothers in Hong Kong also found that parents were concerned about vaccine quality, and they had more trust in immunization products from government sources than from private sources [11]. We did not find any quantitative studies linking perceived quality of vaccine to vaccination decisions in China.

Some participants mentioned that they did not trust their doctor’s recommendation of for-fee vaccines. Wang et al. similarly found that some Chinese parents in Hong Kong distrusted doctors [11], and this lack of trust may be connected with doctors’ wages in China; health care providers earn money from vaccines and drugs prescribed to patients, and so overprescription of drugs, such as antibiotics, is a large problem [30, 31]. However, in their summary of ethnographic material collected in Bangladesh, Ethiopia, India, Malawi, the Netherlands, and the Philippines, Streefland et al. hold that even if a potential vaccine recipient has bad experiences with individual health care workers, he or she may still have trust more generally in the health care system [32], which could explain how Chinese vaccine providers have been able to maintain high coverage of the free, mandatory vaccines.

### Limitations

This study is subject to several limitations. For one, Shanghai is one of the wealthiest and most educated cities in China. Parents in less wealthy and more remote regions may have different underlying beliefs about vaccines which were not found in our study population. Additionally, as with any qualitative study, our study can be used neither to determine the prevalence of any attitude or behavior in the population nor to evaluate the association between attitudes and behaviors. Some benefits, like the herd effect after vaccination, were not explored in this study but could be important in parents’ vaccination decisions. Future studies should use representative samples to quantify some of the preferences that we have remarked on here—particularly through the use of conjoint analyses, which can calculate willingness-to-pay for various attributes of a vaccine. Future qualitative studies could group parents by their vaccine purchasing behaviors (e.g., (1) receiving only free, EPI vaccines, (2) purchasing all for-fee vaccines, (3) using a combination of free and for-fee vaccines, or (4) not receiving any vaccines) to better understand how vaccine concerns vary across parents with diverse beliefs.

## Conclusions

Severity of the disease—particularly in causing long-term disability—emerged as a dominant factor influencing a parent’s decision to get a vaccine. Many parents believed that natural infection was preferable to vaccination, as long as disease was not severe, and many were concerned that imported vaccines were inadequate for Chinese children. Parents’ preferences for vaccines to protect against severe diseases should influence the marketing of vaccines which can protect against different types of diseases. Additionally, vaccination providers can educate parents about the importance of vaccinations in developing an immune system, regardless of the child’s perceived physical constitution. Moreover, future decisions to mandate more vaccines will need to consider how to mitigate potential negative attitudes that Chinese have towards co-administration of vaccines or vaccination against less common or less sever diseases.

## Abbreviations

EPI: Expanded program on immunization
Hib: *Haemophilus influenzae* type b
PCV: Pneumococcal conjugate vaccine
VPD: Vaccine-preventable disease
